# Disclosure to social network members among abortion-seeking women in low- and middle-income countries with restrictive access: a systematic review

**DOI:** 10.1186/s12978-021-01165-0

**Published:** 2021-06-07

**Authors:** Clémentine Rossier, Angela Marchin, Caron Kim, Bela Ganatra

**Affiliations:** 1 Université de Genève, Geneva, Switzerland; 2 Institut National d’Etudes Démographiques, Paris, France; 3 University of Colorado, Denver, USA; 4 UNDP-UNFPA-UNICEF-WHO-World Bank Special Programme of Research, Development and Research Training in Human Reproduction (HRP), World Health Organization, Geneva, Switzerland

**Keywords:** Unsafe abortion, Social network, Access to care, Low and middle income countries

## Abstract

**Background:**

Health care for stigmatized reproductive practices in low- and middle-income countries (LMICs) often remains illegal; when legal, it is often inadequate, difficult to find and / or stigmatizing, which results in women deferring care or turning to informal information sources and providers. Women seeking an induced abortion in LMICs often face obstacles of this kind, leading to unsafe abortions. A growing number of studies have shown that abortion seekers confide in social network members when searching for formal or informal care. However, results have been inconsistent; in some LMICs with restricted access to abortion services (restrictive LMICs), disclosure appears to be limited.

**Main body:**

This systematic review aims to identify the degree of disclosure to social networks members in restrictive LMICs, and to explore the differences between women obtaining an informal medical abortion and other abortion seekers. This knowledge is potentially useful for designing interventions to improve information on safe abortion or for developing network-based data collection strategies. We searched Pubmed, POPLINE, AIMS, LILACS, IMSEAR, and WPRIM databases for peer-reviewed articles, published in any language from 2000 to 2018, concerning abortion information seeking, communication, networking and access to services in LMICs with restricted access to abortion services. We categorized settings into four types by possibility of anonymous access to abortion services and local abortion stigma: (1) anonymous access possible, hyper stigma (2) anonymous access possible, high stigma (3) non-anonymous access, high stigma (4) non-anonymous access, hyper stigma. We screened 4101 references, yielding 79 articles with data from 33 countries for data extraction. We found a few countries (or groups within countries) exemplifying the first and second types of setting, while most studies corresponded to the third type. The share of abortion seekers disclosing to network members increased across setting types, with no women disclosing to network members beyond their intimate circle in Type 1 sites, a minority in Type 2 and a majority in Type 3. The informal use of medical abortion did not consistently modify disclosure to others.

**Conclusion:**

Abortion-seeking women exhibit widely different levels of disclosure to their larger social network members across settings/social groups in restrictive LMICs depending on the availability of anonymous access to abortion information and services, and the level of stigma.

**Supplementary Information:**

The online version contains supplementary material available at 10.1186/s12978-021-01165-0.

## Plain Language summary

Women seeking an induced abortion in LMICs often face inexistent or inadequate, difficult to find and/ or stigmatizing legal services, leading to the use of informal methods and providers, and unsafe abortions. A growing number of studies have shown that abortion seekers contact social network members beyond their intimate circle when seeking care. However, results have been inconsistent. We searched Pubmed, POPLINE, AIMS, LILACS, IMSEAR, and WPRIM databases for peer-reviewed articles published in any language from 2000 to 2018, concerning abortion information seeking, communication, networking and access to services in restrictive LMICs. We screened 4101 references, yielding 79 articles with data from 33 countries for extraction. We grouped countries (or social groups within countries) into four types of settings: (1) anonymous access possible, hyper stigma; (2) anonymous access possible, high stigma; (3) non-anonymous access, high stigma; (4) non-anonymous access, hyper stigma. Most studies fitted Type 3. Disclosing to network members increased across setting types: no women confided in network members in Type 1 settings, a minority in Type 2 and a majority in Type 3. No setting fitted Type 4. The informal use of medical abortion did not modify disclosure to others. Abortion seekers in restrictive LMICs frequently contact their social network in some settings/groups but less frequently in others, depending on the availability of anonymous access to abortion care and the level of stigma. This knowledge is useful for designing interventions to improve information on safe abortion and for developing network-based data collection strategies.

**Supplementary Information:**

The online version contains supplementary material available at 10.1186/s12978-021-01165-0.

## Background

Numerous studies have looked at the links between interpersonal ties and health outcomes [1]. Such effects are both positive and negative, and operate through several pathways: [2] first, emotional support, conflict and practical help directly affect individuals' health resources; support is usually provided by family members or close ties. Second, social norms influence and control individual aspirations and health behaviours, and new normative and behavioural models are acquired through social learning. Third, information and socioeconomic resources are swapped between members of groups sharing similar interests. In the field of sexual and reproductive health, the first two mechanisms—social support and social influence/learning—have attracted attention, because financial costs and sociocultural opposition remain fundamental obstacles to reproductive health-seeking behaviours [3]. For instance, financial transfers within women's close networks matter for their access to prenatal care in Mali [4]. Fears of side effects, fuelled by rumours circulating in social networks reflecting social influence/social learning processes [5] are also identified today as a major obstacles to modern contraceptive access [6].

While a staple of stratification studies, the exchange of strategic information and resources among fellow group members with similar interests—corresponding to Bourdieu's definition of social capital [2]—is less often studied in reproductive health. Yet this mechanism is arguably crucial in women's ability to access (informal) care when engaging in stigmatized sexual behaviours or when experiencing stigmatized health problems (non-marital sexuality, abortion, sexually transmitted diseases, etc.) Adolescents in low-income countries, for example, whose sexuality is a social taboo, turn to their peers for information and advice that is difficult to obtain from authorized sources, and this has prompted the development of peer-led interventions [7]. This pattern reflects the fact that health care for stigmatized reproductive practices in low and middle income countries (LMICs) either remains illegal, as is frequently the case for induced abortion [8], or, when legal, is often inadequate, difficult to find by its target group and/ or stigmatizing, which results in women deferring care or turning to informal information sources and providers [9].

For women seeking an induced abortion in LMICs, access to care is often restricted, leading to unsafe abortions; each year, 97% of the estimated 25.1 million unsafe abortions worldwide occur in the developing world [10]. Unsafe abortion leads to increased health care costs and morbidity, and is a leading cause of preventable maternal mortality, causing at least 22,000 deaths each year [11]. Just half (50.5%) of all estimated abortions in developing countries are safe, compared with 88% of those in more developed countries, a proportion which takes into account issues of underreporting and non-diagnosis [10]. In developed countries, less safe abortions are linked to the use of outdated methods (dilatation and curettage) rather than problems of access: in countries with restrictive laws, women are often able to access services with the help of telemedicine or feminist groups, or by travelling abroad [12]. In developing countries, the expanding informal use of medical abortion (self-administration of mifepristone) in the three last decades has decreased the number of "least safe" abortions in favour of "less safe" abortions [10]. "Least safe" abortions are performed by untrained providers using folk methods (potions, sticks,..); "less safe" abortions correspond to women's self-administration of mifepristone or use of dilatation and curettage by trained providers; "safe" abortions are conducted with modern clinical/ surgical methods (vacuum aspiration) or medical means by medically trained providers.

In the case of abortion, informal advice tends to be unhelpful because the diverse folk recipes which circulate in social networks are generally ineffective [12]; women mainly succeed in obtaining an abortion by visiting an underground or informal abortionist/drug seller who—by definition—is not of public knowledge. So women and their partners (or mothers) turn to their social network to "search for an abortionist", as already shown in a seminal study conducted in the United States before abortion was legalized [13]. To obtain the information they need, women and couples have to *disclose* their situation to their network contacts, to one contact at least; this contact can then continue the search on behalf of an (unspecified) friend [14]. Disclosure of stigmatized behaviours is possible under two conditions: with trusted others (close ties, or weaker ties in the same interest group, such as young people or women) [15] or when the transgression is common to both parties (as with the abortion provider or another abortion seeker) [16].

A growing number of studies have shown that abortion seekers confide in their social network members in order to find informal or illegal abortion services in LMICS where access to abortion services is restricted (i.e. where illegal or informal providers are active). However, results have been inconsistent. In some restrictive LMIC settings, or for some groups, disclosure seems to be very limited, perhaps due to very high levels of stigma [17]. In other settings, especially countries with a mix of formal and formal providers such as India, disclosure to social network members seems to be relatively infrequent [18], for reasons which remain unclear. Moreover, it is unclear how the spread of informal medical abortion in restrictive LMICs has affected disclosure to network members, as the medications and information on how to use them may be easier to find than other types of providers and methods.

This systematic review aims both to identify the restrictive LMIC settings in which women and their partners confide in members of their social networks to obtain information about abortion methods and providers, and to explore possible differences or similarities in information sources between women seeking informal medical abortion and those seeking other abortion methods. As detailed below, we categorize settings into four types by possibility of anonymous (i.e. confidential) access to abortion services and local abortion stigma: (1) anonymous access possible, hyper stigma (2) anonymous access possible, high stigma (3) non-anonymous access, high stigma (4) non-anonymous access, hyper stigma. Studying disclosure to social networks may shed light on the circumstances in which women seek different methods of abortion—safe or less safe—in LMICs with restrictive access, and provide useful knowledge for designing interventions to improve access to information and for developing network-based data collection strategies.

## Methods

We searched Pubmed, POPLINE, AIMS, LILACS, IMSEAR, and WPRIM databases and performed hand-searching for peer-reviewed articles published in any language from 2000 to November 2018 concerning disclosure to network members among abortion seeking women in LMICs where access to safe abortion is restricted. Disclosure to the conjugal or parental dyads, and processes of social support and social influence are outside the scope of the present analysis. Rather, we focus on information sharing across social networks beyond their intimate circle in line with Bourdieu's definition of social capital—the sharing of information and resources in larger groups with common interests and identities[2]—to find abortion providers or methods. An appropriate definition of social network membership in this context [19] may be the people one knows by name, has spoken to in the last year, and could contact if desired [20]. At the other end of the relational continuum, the health professionals that women talk to (school psychologists, health staff at abortion clinics, etc.) are not considered as “social network members,” unless they already belong to the women’s or the couples’ sets of social relations.

In practice, we searched for all LMICs, and removed manually the studies concerning settings with unrestricted access to abortion such as Vietnam or Mexico City. Note that two studies concerning residents of restrictive LMICs travelling to another country with non-restrictive access (Thailand, the United States) were also included, as the abortion seekers relied on their social network to organize their travel and access to care. For all these countries, observational and qualitative studies including women who underwent a medical or surgical abortion (the exact surgical method is not always specified) or an abortion by other methods at any gestational age and which mentioned communication/networking, information seeking or broader terms such as "experiences" or "processes" in the titles/abstracts were screened for inclusion (See Additional file 1 for the search terms).

We reviewed titles and abstracts, and the full article, when necessary, to identify studies for inclusion. Descriptive and qualitative studies were of particular interest, as well as studies using snowball or respondent-driven sampling methods, as these methods imply the existence of social networking among abortion seekers. Interventional studies were considered when attention was given to information seeking, communication, or social support beyond intimate ties.

Study quality was determined using standardized checklists for each study type, and rated from 1 to 5 based on a set number of criteria (different for qualitative and quantitative studies). The most commonly encountered problem was that of missing information on the methods used for qualitative analysis (content analysis should have been mentioned), on data collection or data analysis (such as year missing, demographic data not presented, etc.); in some cases, discussion of biases and limitations was also missing. Studies were downgraded for each problem encountered. A minimum quality level of 3 was set for inclusion in the analysis, corresponding to a level of information sufficient to identify the data biases (for example, only abortion complication patients) so that the review authors could consider it in the analysis even when not mentioned by the study authors.

We did not compute summary measures of association due to heterogeneity in study designs, populations, and outcome measures. We extracted data from the selected studies, initially separating qualitative from quantitative data. More precisely, we extracted all the quotes that referred to how abortion seekers talk to others about abortion in general or disclose to others about this event (to whom, under what conditions, how frequently, for what reasons) and how women who had an abortion are contacted by other abortion seekers. We also extracted quotes about how women get information on abortion services and methods.

We noted in addition all information about the context of abortion for the groups of individuals studied. We documented the following contextual dimensions when the relevant information was provided in the articles:

- Legal status of abortion at the country level;
- Share of illegal abortions;
- Whether abortion services (legal or not) can be accessed confidentially by women on their own, without the help of their community or network (i.e. via flyers or posters, helpline, Internet, or professionals such as pharmacists or healthcare providers, teachers or school psychologists, feminist associations, etc.);
- Level of stigma attached to abortion (mistreatment during abortion or post-abortion care, fear of judgment by others, self-judgement, reaction upon disclosure, etc.)

Based on these dimensions and on the previous literature, we constructed a typology of settings based on two intersecting dimensions: possibility of anonymous (i.e. confidential) access to abortion services and level of stigma. For the first dimension we distinguished settings where anonymous access to services is possible for at least a share of abortion seekers (whether or not these services are legal or safe) versus settings with no anonymous access. For the second dimension we distinguished between hyper stigma and high stigma, hyper stigma being characterized by rejection and hostility on the part of close ties and health staff in response to disclosure; high stigma being characterized by strong disapproval of the practice in general but a relative understanding on the part of close ties and health staff. These two dimensions, when intersected, yielded four possible types of settings:

1. (A share of the) women can access services anonymously but cannot disclose to contacts (hyper stigma)
2. (A share of the) women can access services anonymously and can confide in trusted others in order to find services (high stigma)
3. Women overwhelmingly need help from personal contacts to find services and can confide in trusted contacts in order to find services (high stigma)
4. Women overwhelmingly need help from personal contacts to find services but cannot disclose to contacts (hyper stigma).

In a final step of the analysis, we pooled the extracted content into one description per country. For countries with several studies we sometimes distinguished between different groups (married and not married, immigrants), different regions or time periods when women’s disclosure behaviours or access to abortion services were different. We placed each country/group in one of the four contextual categories, and analysed common patterns of disclosure to network members across types, distinguishing abortion seekers using informal medical abortion from those using other methods.

## Results

**Fig. 1:**
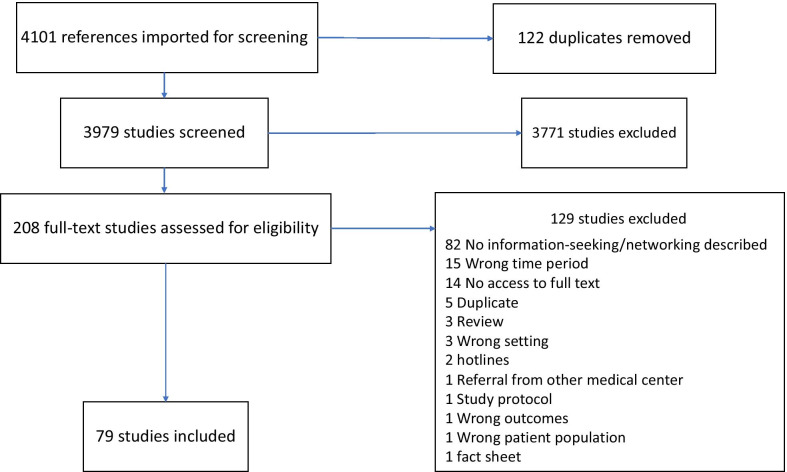
PRISMA diagram (28/11/2018)

The systematic search of Pubmed, POPLINE, and LILACS (see Additional file 1 for search terms), yielded 4101 references, 79 of which were included for analysis (Fig. 1). The AIMS and WPRIM databases were screened by hand, and yielded no additional articles. The IMSEAR database was non-functional during the time of study screening.

The 79 studies selected for inclusion took place in 33 different countries in sub-Saharan Africa (15 countries), Asia [8], Latin America [7], and the Middle East [3], or concerned residents of those countries who sought abortions abroad (Table 1).

**Table 1:** 79 studies in 33 countries

Region/ countries	Studies
Africa	
Ethiopia	Kebede [24], Kebede [23], Alemayehu [46]
South Africa	Orner [38], Orner [37], Gerdts [39], Harries [40]
Nigeria	Bankole [94], Alubo [93]
Zambia	Coast [59], Freeman [60], Cresswell [61], Dahlback [62]
Kenya	Izugbara [63] Osur [64] Mitchell [66] Izugbara [65], Jayaweera [48] Penfold [67]
Ghana	Hill [68] Kumi-Kyereme [69] Aniteye [70], Appiah-Agyekum [71], Rominski [73] Ganle [72], Rominski [74]
Uganda	Nyanzi [75], Atuyambe [76], Marlow [77]
Tanzania	Norris [78], Plummer [79], Rasch [80]
DRC	Rouhani [81], Burkhardt [82]
Burkina Faso	Ouédraogo [83], Rossier [14]
Benin	Baxerres [84] (also Burkina Faso)
Madagascar	Pourette [85]
Botswana	Smith [86]
Malawi	Jackson [95]
Gabon	Hess [96]
Latin America	
Colombia	Brack [25], DePineres [26]
Bolivia	Bury [47]
Brazil	Silveira [22], Arilha [51], Diniz [52], Madeiro [53], Nunes 2013, Heilborn [54]
Chile	Casas [55], Palma Manriquez [56]
Argentina	Ramos [57]
Mexico/California	Grossman [91]
Haiti	Albuja [97]
Asia	
India	Kalyanwala [27], Jeejeeboy [28], Banerjee [29], Banerjee [32], Banerjee [33], Elul [30], Behera [31], Banerjee 2015, Elul [35]
Nepal	Puri [43], Rocca [44], Ohja [41], Andersen [42]
Bangladesh	Messinger [45]
Sri Lanka	Arambepola [50]
Malaysia	Tong 2012
Philippine	Gipson [87]
Cambodia	Petitet [21]
Burma/Thaïland	Arnott [88], Tousaw [89], Tousaw [90]
Middle East	
Iran	Zamanian [36]
Saudi Arabia	Alsibiani [49]
Palestinian OT	Shahawy [98]

Over half of these studies [21] had been published in the previous five years (2014–2018), the rest between 2001 and 2013. More than half of them [22] used qualitative methods (ethnography, in-depth interviews, focus groups) and the rest were based on quantitative analysis of data collected in representative samples (community-level surveys or abortion care patients) or followed a mixed-methods approach (quantitative data from a survey of abortion care patients supplemented by in-depth interviews). All 79 studies met the minimum quality requirement set at 3 out of 5.

We classified all the populations (countries/groups) studied in this meta-analysis into one of the first three configurations of settings. We found no study exemplifying the fourth type of setting (non-anonymous access and hyper stigma), probably because abortions are, by definition, rare in such settings. In these settings women with unplanned pregnancies, and/or their partner or mother, cannot access an anonymous abortion provider since such providers—typically located in a health centre and advertising through flyers or the internet—do not exist; neither can they turn to underground abortion providers, which are probably rare as they need word-of-mouth referral to thrive (and women who want an abortion or have had one do not dare disclose to others because of hyper stigma). In such settings, women with unintended pregnancies likely carry them to term, despite the potential adverse consequences (Table 2).

**Table 2:** 79 studies in four types of settings classed by criteria of anonymous access and stigma

Types	Studies
Anonymous access possible (for a share of women), hyper stigma	Kebede [24], Kebede [23] Brack [25], DePineres [26]
Anonymous access possible (for a share of women), high stigma	Kalyanwala [27], Jeejeeboy [28], Banerjee [29], Banerjee [32], Banerjee [33], Elul [30], Behera [31], Banerjee 2015, Elul [35] Zamanian [36] Orner [38], Orner [37], Gerdts [39], Harries [40] Rocca [44], Ohja [41], Andersen [42], Puri [43] Alemayehu [46] Bury [47] Messinger [45] Petitet [21]
No anonymous access, high stigma	Alsibiani [49] Arambepola [50] Silveira [22], Arilha [51], Diniz [52], Madeiro [53], Nunes 2013, Heilborn [54] Casas [55], Palma Manriquez [56] Ramos [57] Coast [59], Freeman [60], Cresswell [61], Dahlback [62] Izugbara [63] Osur [64], Mitchell [66], Izugbara [65], Jayaweera [48], Penfold [67] Hill [68], Kumi-Kyereme [69], Aniteye [70], Appiah-Agyekum [71], Rominski [73] Ganle [72], Rominski [74] Nyanzi [75], Atuyambe [76], Marlow [77] Norris [78], Plummer [79], Rasch [80] Rouhani [81], Burkhardt [82] Ouédraogo [83], Rossier [14], Baxerres [84] Pourette [85] Smith [86] Tong 2012 Bankole [94], Alubo [93] Jackson [95] Hess [96] Grossman [91] Albuja [97] Arnott [88], Tousaw [89], Tousaw [90] Shahawy [98] Gipson (87)
No anonymous access, hyper stigma	No studies

### Type 1: Anonymous access and hyper stigma

A first configuration of situations concerns countries/groups where abortion seekers speak to no one about what they are going through, except the abortion provider and sometimes a partner or parent (i.e. an intimate tie). They disclose to no one in their larger social network, because of the severe stigma attached to being pregnant /having an abortion in their situation: “family honour”, “suicide of parents”, and “killing a baby,” are terms mentioned by the respondents in the qualitative studies. These women all access (legal or illegal) abortion services anonymously, as services can be approached confidentially.

A qualitative study of *unmarried women in Addis Ababa, Ethiopia* (post-abortion care patients) describes how these women keep their pregnancy secret because of the shame it would bring to themselves and their family. They go to legal abortion clinics in other parts of the city, and if they cannot afford the prices charged, are approached on the parking lot by illegal providers operating nearby. Only one young woman reached out to a community member; she asked a prostitute in her neighbourhood for advice on where to get an abortion [23, 24].

Another qualitative study in *Colombia* describes a set of women who obtained a legal abortion from one of the few legal clinics operating in the capital; these women did not dare speak about their pregnancy and abortion with others because of the very strong anti-abortion stigma. Instead, they used the Internet or referral from community psychologists or teachers, and only occasionally a friend, to find out about the clinics [25]. Another study in Colombia shows that women use the Internet to find addresses of clinics or places to buy misoprostol, and they are approached by illegal providers waiting outside the clinics or sales venues they visit in person. Talking to friends is mentioned with respect to getting information about misoprostol, but it is not clear whether women disclose their pregnancy to obtain it. The women always appear to have gone through the abortion process alone (only one mentions having told her parents) [26].

A qualitative study of *unmarried women in India* (states of Bihar and Jharkhand) who had an abortion (and self-reported it in a survey) shows a similar pattern. Most of these women informed their partner and were supported by him. Some received practical support from an older family member, although he/she tended to respond with hostility, even if the pregnancy was the result of forced sex. Those who told neither their partner nor their family went through the abortion on their own. All these young women used legal or informal services without the help of their social network, because pregnancies outside marriage are strongly condemned in that setting, and services can be found anonymously [27, 28].

The women described in these studies managed to obtain an abortion on their own or solely with the help of a significant other, such as a partner or parent. It seems, therefore, that in these settings women will only have an abortion if they are able to access abortion services without disclosing their situation to other social contacts.

### Type 2: Anonymous access for a share of the women and high stigma

This second type includes settings where abortion seekers only sometimes disclose their secret to trusted friends, relatives, or members of their social group. Two features of the cultural and institutional context seem to explain this pattern of infrequent disclosure. First, abortion is a stigmatized event and women prefer to keep this experience very private if possible. Second, abortion services (whether illegal or legal) are publicly advertised, although not perfectly; only when women cannot find a service provider or method by themselves do they reach out (secretly) to trustworthy or potentially knowledgeable social network members.

Several studies of *married women in India* show that women start by discussing their pregnancy with their husbands and sometimes their in-laws. Most women (or their intimate ties) know where to find legal abortion services. For example, half of the women in a community sample can cite a correct source [29], and a qualitative study mentions that women overwhelmingly know where to find legal services [30]. However, these legal services are often deemed expensive or otherwise inaccessible. Many trained and untrained providers offer illegal services at the local level, and these providers are directly solicited by abortion seekers to obtain information. In one study reviewed, 89% of women who had an abortion in a rural community got their information from local health providers [29], in another study in the slums of Mumbai, 69% of women who had an abortion obtained information on the procedure from local health staff [31]. Women or their closest dyad (partner, in-laws) turn to friends/other community members *only* when they are unable themselves to locate the cheaper and more convenient alternatives to legal options. As a result, most abortion seekers *do not talk* to a friend or community member (in a study of women with abortion complications, only 1 in 5 got advice from a friend) [32–34]. In a community survey of women in the slums of Mumbai, only 19% received help from friends or other women who had had an abortion in the community [31]. For those in need, the help of friends, although only occasional, is nevertheless instrumental in getting an abortion. Another quantitative study in India showed that abortion seekers seldom talk to friends, but that having friends to confide in increases the probability of obtaining an abortion [35]. While sufficient information is lacking to classify the study, married women in *Iran* could be in a similar situation as those in India (relatively easy access to medical staff and limited disclosure to networks), as information about abortion seldom extends beyond the intimate circle [36].

The situation in *South Africa* appears to be analogous. One qualitative study of HIV-positive women who had had a legal abortion describes how abortion services were familiar to many of them. “There was general awareness that free abortions were available in public health sector facilities, gained mostly from local media and clinic posters, but also from knowing other women who had used the services” [37]. (p. 787) (see also [38]). The study underlines women's reluctance to talk to others about their abortion (a taboo much stronger than that of their HIV status), because of the high level of stigma attached to this practice. Another study in South Africa reports that women are relatively knowledgeable about legal abortion services, but fearing the exposure and stigma linked to using legal services they prefer to find alternative solutions [39]. Study participants (all informal sector abortion service users) were found through a respondent-driven-sample (RDS): “44% of participants reported seeking informal sector abortions because they worried someone would find out about the abortion, and 30% reported concerns about mistreatment and stigma from providers in the formal sector”(p.5). Knowledge of these alternative solutions is obtained through friends mainly (63%), but also through posters or flyers (23%). In the group of abortion seekers studied (all informal sector abortions), most women shared their abortion experience with others and knew others who had had an abortion. Similar results were found for women who had had a delayed second-trimester abortion in the public sector [40]. These informal sector or late public-sector experiences contrast with those described by legal abortion seekers in South Africa; on the whole, only a fraction of the women concerned disclose to social networks.

In *Nepal*, abortion became legal relatively recently, but many women still use informal and unsafe services [41–43]. Accordingly, some women still discuss their pregnancy and seek abortion advice from friends or community members. In a quantitative study, 20.3% of women told a friend they were getting an abortion, 25.8% found out how to get an abortion from a friend, and 11.4% reported that a friend was a source of support during the abortion process [44].

Studies in *Bangladesh*, [45] *Ethiopia* [46] (on post-abortion care for patients of any marital status), *Cambodia* [21] and *Bolivia*, [47] while providing limited information on disclosure to network members, seem to indicate similar situations. They suggest that while many women know about and can access abortion services anonymously, not all women want these services or know about them, so turn to friends and community members for information on underground services.

### Type 3: Non-anonymous access and high stigma

In the third set of situations, by far most the most common in the articles analysed, women seeking an abortion, and sometimes their partners/ mothers, overwhelmingly confide in members of their social networks under the cover of secrecy to obtain information about abortion services. Two features of the context seem to explain this pattern. First, abortion is stigmatized, but friends or selected knowledgeable community members are trusted to keep a secret. Second, information about abortion services is not publicly available (whatever the status of the service, legal or not), and health providers who can be approached anonymously do not refer abortion seekers to abortion services. Note that in these settings, women sometimes begin by trying well-known traditional or folk medicine methods on their own. However, these attempts are often unsuccessful, or end in complications that reveal the abortion to many more: for example in the Nairobi slums: “One participant said, ʽI know of a friend who took some Quencher [fruit flavoured drink] that was not diluted and then she bled excessively; by the time she was getting to the hospital, she was dead” [48] (p. 6). Women thus usually need to talk to trusted others to locate an effective method or provider.

Most studies (and countries) described in these articles are in this category. Since women need to talk to trusted others to locate abortion providers or effective methods, and since abortion is stigmatized but accepted by network members, a large majority reach out to their social networks. The only women who do not are those who can access services directly from a health provider (for example their boyfriend is a medical student) and those who have no one in their social network they can trust. The latter can still self-induce the abortion using traditional folk methods, although they are often ineffective and dangerous, as mentioned above. Except in these two situations, trusted network members are consulted to locate “knowledgeable others” (people who know about a method or a provider). Who these “knowledgeable others” are exactly varies across settings, but *women who had an abortion recently* are mentioned in most studies. Other resourceful people cited are *prostitutes, illegal drug sellers, feminist groups, people bringing clients to illegal abortion providers,* and *taxi drivers*. Sometimes these informants are approached directly, even if they do not belong to the seeker's social network; however, this manoeuvre is risky and it is safer when a network member acts as an intermediary.

Under this pattern of information-gathering, a significant share of women in a community will have heard of a network member who has had an abortion, because they have been contacted by them to locate a method or provider. In *Saudi Arabia*, 82% of misoprostol users knew of another user [49]. In *Sri Lanka*, “the majority of [abortion seekers] approached their partners and/or immediate associates to obtain more information about the persons/places available for pregnancy termination (60%) and to accompany them to abortionists (52%)”. According to this study, in the absence of information in their social networks, women in Sri Lanka relied on “unknown sources such as taxi drivers” [50]. In *Brazil*, several studies indicate that women rely on their network to locate where misoprostol can be procured. Who can be reached through these networks is very important, and can change the experience entirely: [22] members of feminist groups were mentioned as especially helpful in this regard, as well as illegal drug sellers, and women who had used misoprostol themselves [51]. “The speed and ease with which a woman triggers a wide network of care and facilities for an abortion is one of the signs of how the culture of abortion is shared among women in Brazil… [Women who had an abortion] assist other women to have an abortion” [52] (p. 1679, translated from Portuguese). A study on Brazilian sex workers also reports that abortion seekers get information on misoprostol from other sex workers, sometimes directly from drug sellers [53]. In another study in Brazil, 30 teens having post-abortion care were interviewed: 80% got their information on where to buy Cytotec from friends; they consulted the Internet to find out *how* to take the drug. Note that Misoprostol is not the main method everywhere in Brazil, and in some cases, economically advantaged women do not need the help of their networks to get safe (albeit illegal) abortions from abortion clinics [54].

In *Chile*, most abortion seekers also appear to rely on their friends to locate the abortion drug, but some women manage to find misoprostol on the Internet by themselves: “The sellers are normally searched for online or through a friend or acquaintance who previously bought the pills or knows a seller… A participant who had an abortion five years prior to the interview now volunteers to help and support other women” [55]. A study on a sample of 30 university students obtained by snowball sampling states: “Medical abortion […] was common knowledge among these young women, who directly or indirectly knew of other young women who had used it. This knowledge circulates within young women's circles, despite being ‘forbidden’”. Friends connect abortion seekers to health providers or feminist groups for exact information on where to obtain and how to use drugs for medical abortion [56].

In a qualitative study of women who had used medical abortion in *Argentina*, networking was very important in deciding to proceed and in obtaining the necessary medication. While the Internet and feminist groups helped (two women bought their product directly on the Internet), the fact that a prescription was needed made things difficult. “The complexity of this endeavour depended on getting the prescription needed to purchase it; having contacts who could identify which pharmacy to go to; finding a pharmacy that would sell it; having enough money; and getting the support of friends and/or family”[57] (p.7).

Although abortion is legal to a certain extent in *Zambia* (more precisely, abortion policies are ambiguous and the provision of legal abortion care is low) [58], research indicates that women obtain legal abortion services largely thanks to the help of their network. A few women who have no one to confide in end up having the most dangerous abortions and make many attempts with improbable methods; conversely, women who happen to know a health provider get very rapid access to safe abortion [59, 60]. Many women thus know about someone who has had an abortion: in a random sample of women “21% knew of at least one person who had had an induced abortion over the past 5 years” [61]. (p.3) In a study of young Zambian women presenting to a hospital with abortion complications, some quotes indicate that girls were guided by friends to a method, but other women seem to resort to self-administered folk methods to avoid talking to others, with their attempts ending in complications [62].

In *Kenya*, while a few hospitals offer safe, legal procedures, they are expensive, and women fear being stigmatized and exposed to the public eye if they use them. Consulting friends can be helpful for obtaining an anonymous abortion, as is illustrated by the following quote: “One respondent offered: ‘I went to a Traditional Birth Attendant because she had helped some people I know and she keeps secrets. I did not even know she provides abortion services to women. It was a friend that she helped who directed me to her’ˮ [63] (p.14). Another study undertaken in Kenya (with interviews of illegal providers) shows how former abortion clients are key links to future clients for abortion providers: “A clandestine abortion provider indicates: ʽI get at least three cases per month, mostly one per week. They are mostly school girls. They are referred by those I have treated beforeʾ” [64] (p.36). The study also confirms that prospective abortion seekers talk to a large range of people in their network before securing access to abortion (see also [48, 65]). For example, in a study of 614 students in Nairobi, almost half (45%) of the young people in the study knew peers who had had abortions [66]. But in a study with 22 women who attended six private clinics (abortion and post-abortion care) offering legal abortions in Western Kenya, only some of the women relied on friends to find methods; others were referred directly to the clinics by health staff or pharmacists [67]. These parts of Kenya are more similar to type 2.

Like Kenya, *Ghana* is a country where safe, legal abortion services have been difficult to access for a long time, so many women relied heavily on friends until recently. “Friends were the people most often informed about planned abortions and were contacted because they may know of a good and inexpensive method, are trusted to keep the abortion a secret, and to help if anything goes wrong”[68] (p. 2019–20). Moreover, abortion seekers in Ghana appear to consult quite a few people even before deciding to terminate, when they are unsure what to do about the pregnancy [69]. Recently, some legal abortion services have become available in Ghana, so some populations living near these services can be labelled as type 2, as some women get an abortion directly without telling others [70–72]. In another study we see that women using medical abortion informally do so with the help of their networks solicited in secret, although this does not seem to be the case for women using legal services [73]. In a study of university students, 42% knew someone who had had an abortion [74].

An in-depth qualitative study of young taxi drivers in *Uganda* sheds light on how they help women and couples get abortions, and on how younger people in general help each other to circumvent the strict demands of the older generations and the authorities with regard to morality and sexual conduct [75]. Another study on adolescents in Uganda shows that young people know about others who are seeking an abortion since they talk about them in focus groups [76]. In a study on sex workers in Kampala, all of the them consulted their fellow sex workers, friends, peer educators or community outreach educators when deciding how and where to obtain an abortion [77].

In a study in *Tanzania*, on the Island of Zanzibar, researchers were very successful in implementing a RDS strategy for abortion seekers “Despite the stigma of abortion, women talk with social contacts about abortion, and thus can connect each other via chain-referral sampling” [78] (p.7). Investigators were also able to get many detailed accounts of the abortion experience of friends in a number of villages in mainland Tanzania [79] (see also [80]). In the *DRC* likewise, RDS was used to interview a sample of women survivors of war and sexual violence, some of whom terminated a pregnancy that resulted from rape. The study shows that for these women, social networks were valuable in finding an abortion method. The snowball sampling strategy also suggests that women are aware of others in their networks who have undergone abortion [81, 82].

In *Burkina Faso*, a study provides qualitative evidence of such mechanisms and uses it to collect quantitative data on abortions among the respondents' trusted others [14]. A qualitative study in the same country confirms the key role of friends in securing abortion services, although one resourceful woman (a law student) obtained misoprostol directly from health personnel by arguing her case [83] (see also [84]). These mechanisms are confirmed for *Benin*, where friends or relatives are always used to locate one of the numerous small private health centres far from home where the abortion can be done discreetly [84]. Women in *Madagascar*, younger women especially, consult their friends about abortion. They also consult their network of neighbours/personal contacts who are medical professionals, either when they decide to terminate a pregnancy or if complications arise [85]. In *Botswana*, a qualitative study found that “most of the providers are women, and the process of finding an illegal practitioner and accessing the service operates through a women-only system of information: ‘you’ll always find someone who knows someone who can help you’”[86] (p. 46). This is also the case in *Malaysia* [86] and in the *Philippines *[87].

A study of border residents of *Myanmar* (where abortion is strongly prohibited) who travelled to Thailand (where access to abortion services is legal on health grounds) depicts a similar context. Not all of these women can access legal services on their own and some go to informal providers on either side of the border with or without the help of friends [88]. Burmese migrants also need their friends to navigate the complex and unfamiliar Thai system of legal abortion [89, 90]. Similarly, in a study of 17 *Mexican* women who crossed the border to use legal abortion services in California, the women reported learning about the clinic from family members, friends, or the Internet. In some cases, they or their partners had previous experience of these services [91].

The remaining studies provide less detailed results, but similar situations appear to exist in a number of other countries, as in Malysia [92]. In *Nigeria,* older studies, while fragmentary, depict a situation where most women disclose to others in order to find abortion methods or providers [93, 94]. The situation today may be closer to type 2, as in Ghana or Kenya. The situation appears to be similar for *Malawi* (safe abortion for those with good connections) [95]. In *Gabon*, a small study indicates that in most cases women turn to friends to locate a method or provider [96]. In *Haiti,* providers operate clandestinely and the topic remains secret, but focus group participants mention friends who have had abortions [97]. In the *Palestinian Occupied Territories,* 4 out of 10 respondents (40 total, general populations) know a friend who has had an abortion. A range of options to terminate exist but seem difficult to access [98].

## Discussion

The likelihood for women of using their networks and confiding in trusted others to locate abortion providers appears to be related to the legal status of abortion. Settings where women can access abortion services anonymously (and thus do not need to talk to their friends) (Types 1 and 2) are usually also those where abortions are allowed, on at least some grounds. But not all settings where legal abortion exists offer “anonymous” access to these services, Zambia being a striking example (Type 3). Moreover, in countries where abortion is legal, many or most women may refuse to use these services, deemed as too costly or too far away, not sufficiently confidential, or stigmatizing, as in South Africa or India, and more recently in Kenya or Ghana (Type 2). A varying share of women (but not the majority) consult their networks for information on alternative services in these countries. On the other hand, countries where abortion is illegal and safe services are unavailable, anonymous access to services may still exist thanks to the Internet, as is the case in Bolivia, which is similar in this respect to high-income restrictive countries.

Informal self-use of medical abortion does not seem to be systematically associated with more or less information sharing. In some cases, like in Bolivia (also documented in Chile), information on the Internet is sufficient to obtain and self-administer this method, so abortion seekers can avoid asking their networks for help (as in high-income countries with restrictive abortion laws). But in other places, like India or the Philippines, using this method informally requires more assistance from trusted network members and knowledgeable others, because women do not know where to buy the drug and how take it, unlike women who go to a publicly known (albeit often also informal) surgical abortion provider.

Another interesting finding is that disclosing to trusted network members is not sufficient to find abortion services directly: these contacts often go on to locate knowledgeable informants in the community. These resourceful informants are women who have had an abortion themselves, or health workers. In some settings, sex workers, taxi drivers, illegal drug sellers, people working with illegal providers and members of feminist groups are key informants in the search for an abortion provider. Part of the information sharing will converge towards these individuals. They are not part of women's networks, but have usually helped a network member (or a friend of a network member) in the past.

This study has a number of strengths. Having multiple team members to perform the data analysis contributed to internal validity. Multilingual team members also improved the access to data published in languages other than English. A limitation of this study is that much of the data is based on self-reported information, and is subject to bias, especially given the sensitive nature of the topic. Moreover, there is no recognized model for collecting data on information-sharing or disclosure behaviours of abortion seekers, so the studies reviewed here seldom collected enough data. They all document the sharing of abortion information between abortion seekers and their networks. While disclosure can be assumed in most cases, this is not always explicitly stated. When disclosure is explicitly documented, these studies usually distinguish the person spoken to (partner, mother or other relative, friends, health personnel), but precision is sometimes lacking. The exact type of information shared is not always clear either. Similarly, the exact conditions under which disclosure is taking place (degree of trust, secrecy) is rarely specified. While the incomplete nature of the information on networking in these studies is a real limitation to our classification, the fact that countries were often covered in multiple studies helped construct a typology of disclosure for countries or groups within countries.

Comprehensive data collection on the topic could include the following topics: (1) The extent to which information on access to abortion services is public (i.e. how confidentially abortion services can be accessed: ads, Internet, health providers as a source of information) and how acceptable publicly available options are to women (stigmatization during care, privacy, costs). (2) The degree of abortion stigma: will close friends and relatives be understanding when told in secret about a pregnancy to be aborted? (3) General information about abortion overheard or obtained from others should be distinguished from disclosure to others to obtain more precise information. (2) In case of disclosure, with whom precisely the abortion was discussed should be documented: partner, mother/father or tutor or mother/father in-law; friends; other relatives (cousins etc.); other network members (specify condition of trust). (4) Whether these network members had an abortion themselves should also be documented. (5) Whether these network members helped in accessing services directly or whether they had contacted someone else, and whom (specify conditions of trust).

## Conclusion

It is clear that social networking is taking place among women and couples seeking abortion in restrictive settings, although to varying degrees depending on the context. A better understanding of information-gathering behaviours could inform the development of programmes that can provide accurate information about safer abortion options. Regarding adolescent contraceptive use, recent reviews shows that peer-led interventions were poor at changing behaviours, but succeeded in conveying information and increasing reproductive health knowledge [7, 99]. As access to the right information is key to obtain a safe abortion in a restrictive setting, [59] there may be some scope for peer-led interventions to improve access to legal and safe abortion care. Also, given the evidence to show that social networking exists among women who seek abortions in restricted settings, this network could be utilized to inform future sampling strategies for outlining the sociodemographic characteristics of abortion seekers and safety of abortions in these settings. Conversely, these results also underline that network sampling methods are not suitable for documenting abortion practices in a representative manner in restrictive low and middle-income countries where a large share of women access abortion services anonymously, without the help of network members beyond their intimate circle.

## Supplementary Information

**Additional file 1:** Search strategy.

## Data Availability

Not applicable.

## Abbreviations

LMICs: Low- and middle-income countries
